# Topics of the Month

**Published:** 1896-01

**Authors:** 


### TOPICS OF THE MONTH.

It is high time that an attempt was made to regulate by law the giving of medical expert testimony in the courts. A recent Albany dispatch states that an important measure of this kind will be introduced into the legislature during the coming session. Heretofore any physician, no matter how shallow his knowledge, has been called upon to testify in important cases where even life was at stake. It is now proposed to abolish expert medical testimony in this state, in so far as it is now given by physicians of any degree of ability who may be called by the lawyers in a trial case.

As a substitute for this time-honored but dangerous fashion several prominent members of the medical profession, whose names cannot at this time be mentioned, have drawn a bill which will be very radical in its terms and will lodge the right to give medical expert testimony in a nonpartisan board appointed by the Governor, by and with the advice and consent of the Senate. The province of the board, according to a telegraphic synopsis of this bill, will be to act in the capacity of instructors and advisors to the courts. It will be large enough to provide amply for all criminal trials and the quorum will be small enough to allow sections of the board to act with the courts in various localities.

The members will be chosen from the ranks of the most expert alienists, having a high ascertained qualification. The function and power of the board will be to examine with reference to the sanity or insanity of any accused person and will have the power to call for any evidence throwing light upon the case. When any section of the board disagrees upon the questions involved, the whole board may be called together for a decision. Under no circumstances will any physician not a member of the board be allowed to testify in any of the courts of the state unless it be upon the absolute facts of the trial.

Any such expert may, however, appear before the board and give evidence. The matter of remuneration of the board has not yet been decided, and the bill may be amended so as to make the board consist of the medical heads of each of the state hospitals for the insane in the state.

This change is so radical that the proposed measure will be sure to call forth a great deal of discussion, hence we devote considerable space to an abstract of the bill.

---

Electric railway ambulances, as we have heretofore shown, are very much needed in Buffalo. Let the Buffalo Traction Company announce, in addition to the various improvements that it has already proposed to make in our street railway service, should it obtain a charter, that it will place trolley ambulances in service as soon as its road opens. It will thus show its readiness to adopt any and all improvements that are needed so much in Buffalo.

We affirm, notwithstanding the many assertions to the contrary, that no city in the Union of the size of Buffalo has as inefficient street railway service. It is only a short time since straw was abolished in the cars and a still shorter period since the first car was heated in winter. It is astonishing what a disposition there is here for the citizens to plant themselves across the line of progress. But we have observed that the chief opposition to additional street railway service, as proposed by the Buffalo Traction Company, comes from people who ride in carriages.

---

THE special committee of National Guard surgeons, which the Surgeon-General appointed at the September meeting, held a spirited session in the 71st Regiment Armory, Monday, November 11, 1895. Major Herman Bendell, of Albany, presided, but Captain Burr, the secretary of the committee, was absent. The discussions which took place showed that the various members had given the matter of improvement of the medical department much thought since their appointment, and that they were harmonious on matters of most importance. But no report to the Surgeon-General was formulated. Many recommendations were agreed upon, among which the most important were the equipment of the guard with ambulances, the enlargement of hospital corps and the formation of regimental ambulance corps. But these matters and all others were recommitted to a sub-committee, of which Major Stimson, of the 7th Regiment, was made chairman, and this committee will make the final report to the Surgeon-General. The department is sorely in need of funds, and this fact will be made clear to the Commander-in-Chief through the Surgeon-General.

---

IT IS announced that an anti-noise league has been organised at Phenix, Arizona. Commenting upon this the *Maryland Medical Journal* says :

> If noise disturbs places of that size, there is no wonder that the inhabitants of large cities complain against the clamor and the crying of hucksters, the clanging of bells and the rattling of vehicles of all kinds. Much of this noise is necessary, but some of it can be avoided and especially the ringing of bells and the blowing of whistles. Baltimore has a law forbidding steam railroad engines from blowing within the city limits, but the steamboats and the factories keep it up at certain hours at a deafening rate. In the morning, at lunch time and in the evening, the large factories blow for what seems to be a very long time, and then the clocks, not all being alike ; one whistle succeeds another, until for ten or fifteen minutes, at least four times in the day, these sounds are repeated.

In Buffalo the noise nuisance seems to be increasing rather than diminishing, notwithstanding the fact that numerous appeals have been made to the authorities asking that unnecessary and extreme noises be restrained.

---

The *Comité Franco-Américain*, of Paris, has issued a pamphlet for the purpose of promoting the interest of American students in France. An advisory committee has also been formed in the United States, under the presidency of Professor Simon Newcomb, of Washington. In the pamphlet referred to is published a memorial by Professor Harry J. Furber, Jr., of Chicago, which was presented to the ministry of public instruction at Paris. Mr. Furber finished his college courses in the United States and then enrolled himself as a student at the German universities. He received the degree of Ph. D., at Halle, and then went to Paris, where he soon found that the French institutions of learning possessed all that is needed by American students who are desirous of completing their literary or scientific training by two or three years of post-graduate study.

This pamphlet, together with much other information on the subject, can be obtained by addressing M. Paul Melon, General Secretary of the Committee of Patronage for Foreign Students, 24 Place Malesherbes, Paris, France.

---

Commenting upon the resignation of Dr. Cyrus Edson and the expulsion of Dr. Edward C. Mann from the Medical Society of the County of New York, the *Journal of the American Medical Association*, in its issue of December 7, 1895, makes the following astonishing statement :

> It would appear that there has been sudden and unlooked for stimulation of the ethical conscience of a body that has for ten years or more been in affiliation with the "no code" state medical society. We trust that the change of heart is deep, sincere and not based on any alleged approaching bankruptcy of the New York profession. . . . . The quickening of ethical conscience in the society, whereby professional honor is appealed to by many who have heretofore objected to any ethical rules, shows clearly enough the absolute necessity for rules of some sort. "No code" may be extremely interesting as a theory, but it invariably has commercialism as its underlying basis.

This proved a sufficient reason for the *Philadelphia Polyclinic*, in its issue of December 14, 1895, to propound the following conundrums, which, in the vernacular of Dr. De Wolf Hopper Syntax, may be termed "corkers":

We should like to ask the *Journal* in all sincerity the following questions: First, is the signing of certificates of nostrums by a member of the New York County Medical Society, a no code institution, more culpable than similar action upon the part of a member of the American Medical Association? Second, is there any provision in the constitution or rules of the American Medical Association by which one of its officers who violates its code of ethics can be disciplined by the association itself in the absence of action upon the part of his local society?

We may with propriety add: Is it a greater moral crime against ethics to be a member of the Medical Society of the State of New York than to advertise in the various journals that one is prepared to go to any part of the county to do any sort of professional consulting or surgical work? We wait with some interest the *Journal's* answer to these pertinent questions.

